# Haplotypes of NOS3 Gene Polymorphisms in Dilated Cardiomyopathy

**DOI:** 10.1371/journal.pone.0070523

**Published:** 2013-07-29

**Authors:** Lova Satyanarayana Matsa, Advithi Rangaraju, Viswamitra Vengaldas, Mona Latifi, Hossein Mehraban Jahromi, Venkateshwari Ananthapur, Pratibha Nallari

**Affiliations:** 1 Department of Genetics, Osmania University, Hyderabad, Andhra Pradesh, India; 2 Institute of Genetics and Hospital for Genetic Diseases, Begumpet, Hyderabad, Andhra Pradesh, India; Morehouse School of Medicine, United States of America

## Abstract

Dilated Cardiomyopathy (DCM) is characterized by systolic dysfunction, followed by heart failure necessitating cardiac transplantation. The genetic basis is well established by the identification of mutations in sarcomere and cytoskeleton gene/s. Modifier genes and environmental factors are also considered to play a significant role in the variable expression of the disease, hence various mechanisms are implicated and one such mechanism is oxidative stress. Nitric Oxide (NO), a primary physiological transmitter derived from endothelium seems to play a composite role with diverse anti-atherogenic effects as vasodilator. Three functional polymorphisms of endothelial nitric oxide synthase (NOS3) gene viz., T-786C of the 5′ flanking region, 27bp VNTR in intron4 and G894T of exon 7 were genotyped to identify their role in DCM. A total of 115 DCM samples and 454 controls were included. Genotyping was carried out by PCR -RFLP method. Allelic and genotypic frequencies were computed in both control & patient groups and appropriate statistical tests were employed. A significant association of TC genotype (T-786C) with an odds ratio of 1.74, (95% CI 1.14 - 2.67, p = 0.01) was observed in DCM. Likewise the GT genotypic frequency of G894T polymorphism was found to be statistically significant (OR 2.10, 95% CI 1.34–3.27, p = 0.0011), with the recessive allele **T** being significantly associated with DCM (OR 1.64, 95% CI 1.18 - 2.30, p = 0.003). The haplotype carrying the recessive alleles of G894T and T-786C, C4bT was found to exhibit 7 folds increased risk for DCM compared to the controls. Hence C4bT haplotype could be the risk haplotype for DCM. Our findings suggest the possible implication of NOS3 gene in the disease phenotype, wherein NOS3 may be synergistically functioning in DCM associated heart failure via the excessive production of NO in cardiomyocytes resulting in decreased myocardial contractility and systolic dysfunction, a common feature of DCM phenotype.

## Introduction

Dilated cardiomyopathy (DCM) is characterized by left ventricular dilatation and systolic dysfunction in the absence of associated conditions like hypertension, valve disease or coronary artery disease sufficient to cause global systolic impairment. It is one of the most frequent causes of heart failure with systolic dysfunction and is the leading indication for heart transplantation. The major causal genes implicated in the pathology of DCM are sarcomeric and cytoskeletal gene/s. However, modifier genes and environmental factors are also considered to play a significant role in variable phenotypic expression of the disease, hence identification of the modifier gene/s may complement the findings on causative gene/s.

Dilated cardiomyopathy can also result due to oxidative stress, wherein nitric oxide (NO) seems to play a key role. NO is a primary physiological transmitter derived from the endothelium, and plays a composite role with diverse anti-atherogenic effects as vasodilator [1]. NO is synthesised from L- arginine by means of endothelial Nitric Oxide Synthase (eNOS, type III), an isoform of Nitric Oxide Synthase (NOS) which is predominant in the walls of the blood vessels. Endothelium-derived nitric oxide (eNO), synthesized by eNO synthase (eNOS encoded by NOS3 gene), plays a key role in regulating vessel wall function and cardiovascular homeostasis. Apart from endothelial cells, eNOS is expressed in cardiac myocytes, hence eNOS is the key isoform participating in mechanosensitive regulation of cardiac function [2].

The endothelial nitric oxide synthase gene localized to 7q 35-36, comprises of 26 exons spanning 26 kilo bases and encodes an mRNA of 4052 nucleotides. The NOS3 gene harbors many polymorphic sites including SNPs, variable number tandem repeat (VNTR) sequences etc. The most examined and functionally related polymorphisms are T-786C of the 5′UTR region, G894T in exon7 and 27 bp VNTR polymorphism in intron 4 respectively [3], [4].

Variable expression of endothelial nitric oxide synthase (NOS3) enzyme due to NOS3 gene polymorphisms have been reported to be a significant contributor to cardiovascular morbidity and mortality [5]. Studies on myocardial expression of NOS revealed an upregulation of NOS3 expression in patients with heart failure, an end stage of DCM [6]. These findings have demonstrated the increased NO production as an important component in heart failure [6], and the impact of the NOS3 variants further supports a significant regulatory role for NOS3 [7]. The sequence variation in the NOS3 gene might influence nitric oxide production, and thereby affect the progression of DCM. The role of these polymorphisms in DCM as one of the modifier gene still needs to be elucidated to understand the pathogenesis of dilation of the ventricles. Hence the study aims to evaluate the role of NOS3 polymorphisms in DCM manifestation.

## Results

### Genotype and Allele Frequencies of NOS3 Polymorphisms

The genotype and allele frequency distribution for the three SNPs are represented in Table 1&2, respectively. Odds risk estimate test was carried out to estimate the risk of the allele and genotype frequencies in patient group compared to the controls (Table 3).

**Table 1:** Genotypic frequency distribution of NOS3 polymorphisms in Controls and DCM groups.

SNP ID	Gene location	Genotype	Controls (N = 454)	DCM (N = 115)	#χ^2^ (p value)
rs2070744	−786	TT	284 (0.62)	58 (0.51)	6.898 (0.03)*
		TC	146 (0.32)	52 (0.45)	
		CC	24 (0.05)	5 (0.04)	
27 bp_VNTR	Intron 4	4b/4b	308 (0.67)	78 (0.68)	0.203 (0.90)
		4b/4a	135 (0.29)	35 (0.30)	
		4a/4a	11 (0.02)	2 (0.017)	
rs1799983	Exon 7	GG	314 (0.69)	61 (0.53)	11.149 (0.003)*
		GT	108 (0.24)	44 (0.38)	
		TT	32 (0.07)	10 (0.09)	

# Pearson chi square value with degree of freedom = 2.

**Table 2:** Allelic frequency distribution of NOS3 polymorphisms in Controls and DCM groups.

SNP ID	Gene location	Alleles	Controls (N = 454)	DCM (N = 115)	#χ^2^ (p value)
rs2070744	−786	T	0.79	0.73	2.977 (0.08)
		C	0.21	0.27	
27 bp_VNTR	Intron 4	4b	0.827	0.83	0.0004 (0.98)
		4a	0.172	0.17	
Rs1799983	Exon 7	G	0.81	0.72	8.28 (0.004)*
		T	0.19	0.28	

# Pearson chi square value with degree of freedom = 1.

**Table 3:** Odds risk estimation of NOS3 polymorphisms in DCM compared to controls.

SNP ID	Genotypes compared	Controls	DCM	OR (95% CI)	p value
rs2070744	C vs T	194 (21%)	62 (27%)	1.36 (0.97–1.89)	0.07
	TC vs TT	146 (32.2%)	52 (45.2%)	**1.74 (1.14–2.67)**	0.01*
	CC vs TT	24 (5.3%)	5 (4.3%)	1.02 (0.37–2.78)	0.96
	TC/CC vs TT	170 (37.4%)	57 (49.6%)	**1.64 (1.09–2.48)**	0.018*
27 bp_VNTR	4a vs 4b	157 (17%)	39 (17%)	0.97 (0.66–1.43)	0.90
	4b4a vs 4b4b	135 (29.7%)	35 (30.4%)	1.02 (0.65–1.60)	0.91
	4a4a vs 4b4b	11 (2.4%)	2 (1.7%)	0.72 (0.16–3.30)	0.67
	4b4a/4a4a vs 4b4b	146 (32.2%)	37 (32.2%)	1.00 (0.65–1.55)	0.99
rs1799983	T vs G	172 (19%)	64 (28%)	**1.64 (1.18–2.30)**	0.003*
	GT vs GG	108 (23.8%)	44 (38.3%)	**2.10 (1.34–3.27)**	0.0011*
	TT vs GG	32 (7%)	10 (8.7%)	1.61 (0.75–3.44)	0.22
	GT/TT vs GG	140 (30.8%)	54 (47%)	**1.99 (1.31–3.01)**	0.0013*

OR – Odds Ratio, CI – Confidence Interval.

#### T-786C polymorphism (rs2070744)

The C allele frequency was found to be high in DCM group (27%) compared to the control group (21%). TC genotypic frequency was found to be predominant in DCM group (45%) compared to controls (32%) with the difference being statistically significant (p = 0.03). The TC genotype was found to be associated with 1.74 folds increased risk for DCM compared to TT genotype (OR 1.74, 95% CI 1.14–2.67, p = 0.01). Based on the dominant model, combination of TC+CC genotypes were also observed to be associated with high risk for DCM (OR 1.64, 95% CI 1.09–2.48, p = 0.018), further strengthening the association of ‘C’ allele with DCM manifestation.

#### G894T polymorphism (rs1799983)

The frequency of T allele was found to be predominant in DCM group compared to controls (28% vs 19% respectively), with a 1.68 folds increased risk for DCM (OR 1.64, 95% CI 1.18–2.30, p = 0.003). Heterozygotes (GT) were found to be predominant in the DCM group compared to controls (38%, 24% respectively, p = 0.003) with 2 folds increased risk for DCM, which was statistically significant (OR 2.10, 95% CI 1.34–3.27, p = 0.0011). Based on the dominant model, combination of GT+TT genotypes were also observed to be associated with high risk for DCM (OR 1.99, 95% CI 1.31–3.01, p = 0.0013), further confirming the risk of ‘T’ allele in DCM.

#### 27 bp_VNTR Intron 4 polymorphism

The frequency of 27 bp_VNTR Intron 4 genotypes was found to be almost similar in both controls and DCM groups, and hence the odds ratio was found to be insignificant. The above findings clearly point towards the combined synergistic effect of the various SNPs of NOS3 gene in DCM onset.

### Linkage Disequilibrium

**Figure 1:**
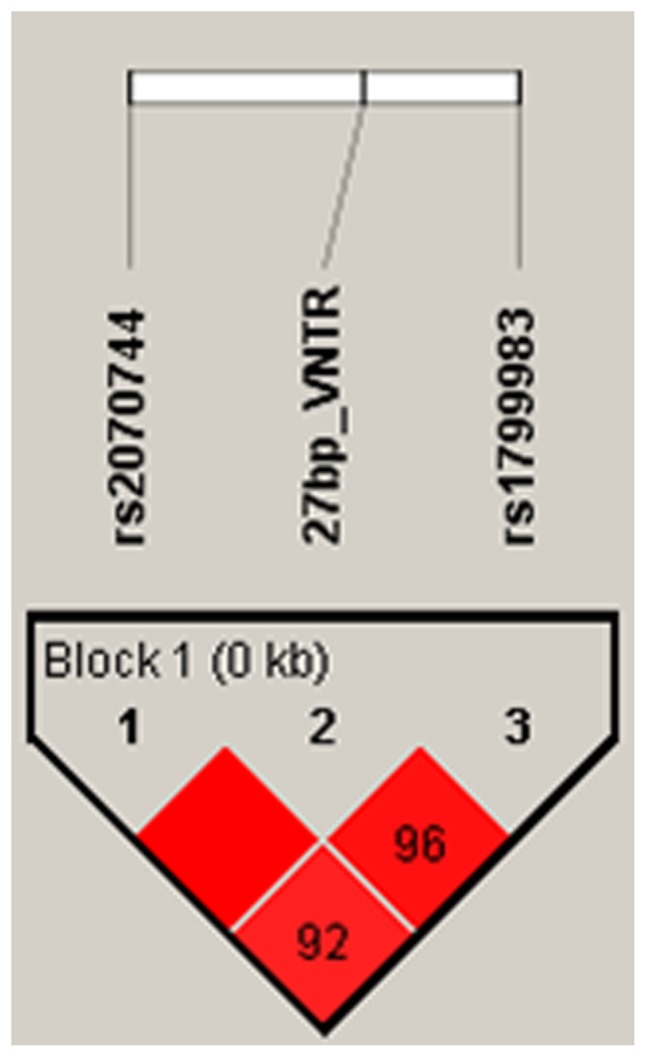
Linkage Disequilibrium plot for NOS3 polymorphisms in cases and controls: data is D′ values.

The extent of linkage disequilibrium (LD) was expressed in terms of the maximum likelihood estimate of disequilibrium, D′ and as per the LD plot (Fig. 1), the loci combinations T-786C:27 bp_VNTR and 27 bp_VNTR:G894T were in perfect LD (D′ = 0.99, 0.96 respectively). Pairwise LD estimates were also calculated for the three polymorphisms studied (Table 4).

**Table 4:** Pairwise Linkage Disequilibrium estimates in DCM compared to controls.

Locus pair	D′	LOD	r^2^
T-786C : 27VNTR	0.99	0.13	0.84
T-786C : G894T	0.92	0.15	0.88
27VNTR :G894T	0.96	0.13	0.85

### Haplotype Analysis

Haplotype analysis is believed to be more informative approach in strengthening the genetic influence on disease manifestation than testing for individual genotypes, hence haplotypes were constructed based on the three polymorphisms and analyzed for the possible association with DCM. Of the five haplotypes obtained (Table 5), two haplotypes carrying the recessive allele of G894T polymorphism, C4bT and T4bT were found to be significantly predominant in the disease group. The C4bT haplotype was found to be predominant in DCM than controls with a 7.37 fold significant increase, (OR 7.37, 95% CI 3.51 - 15.50, p<0.0001). The other haplotype T4bT was also found to exhibit a significant increase of 3.42 fold in DCM (OR 3.42, 95% CI 1.05 - 11.21, p = 0.042). Hence C4bT & T4bT haplotypes could be the risk haplotypes for DCM, with the ‘T’ allele of G894T contribution being significant in risk stratification of DCM.

**Table 5:** Haplotype frequency distribution among controls and cases.

S. No	rs2070744	27bp_VNTR	rs1799983	Controls	DCM	OR (95% CI)	P-value
1	T	4b	G	0.777	0.708	1.00	–
2	C	4a	T	0.170	0.166	0.81 (0.52 - 1.26)	0.35
3	**C**	**4b**	**T**	**0.014**	**0.086**	**7.37 (3.51 - 15.50)**	<0.0001*
4	C	4b	G	0.026	0.014	0.56 (0.16 - 1.90)	0.35
5	**T**	**4b**	**T**	**0.009**	**0.023**	**3.42 (1.05 - 11.21)**	0.042*

OR – Odds Ratio, CI – Confidence Interval.

## Discussion

Among the many polymorphisms of the NOS3 gene, it is now evident that the intron 4b/4a, the G894T, and the T786C variants have important implications in the etiopathogenesis of cardiovascular diseases [8], [9], [10], [11], [12] and DCM being a complex disorder associated with heart failure may be one such CVD, with the underlying mechanism being oxidative stress. NOS3-deficient mice have also shown increased LV dilation, hypertrophy, and mortality rates when compared to littermate controls, suggesting the role of NOS3 in LV remodeling [13]. Hence in the present study, an attempt is made to evaluate the NOS3 polymorphims in DCM etiopathogenesis.

In case of T-786C polymorphism, heterozygotes were found to be predominant among DCM group compared to controls (45% vs 32%), with a 1.74 folds increased risk (OR 1.74, 95% CI 1.14–2.67, p = 0.01). According to Nakayama *et al.,* [12] the −786C allele was found to be associated with a significant reduced NOS3 promoter activity, thus leading to the decreased production of NO, a hallmark of oxidative stress mechanism associated with DCM.

The T allele of G894T polymorphism was found to be significantly associated with DCM (OR 1.64, 95% CI 1.18–2.30, p = 0.003). Indeed, predominance of homozygotes (TT) & heterozygotes (GT) was found in DCM than controls (47% vs 31%), and the difference being statistically significant (OR 1.99, 95% CI 1.31–3.01, p = 0.0013). The T894 variant was known to be associated with poorer event-free survival in nonischemic cardiomyopathy [7]. Hence this variant may influence the severity of the disease leading to heart failure. In the present study, the risk to DCM is about 1.64 fold higher in subjects carrying the T allele of G894T polymorphism, hence such observation is justifiable.

Further, no significant association of the 27 bp_VNTR polymorphism with DCM was observed in the present study. This is in accordance with the findings of Ozhan et al [14].

Nitric oxide derived from eNOS plays an important role in cardiomyocyte proliferation and maturation during early neonatal heart development [15]. The NOS3 is also constitutively expressed in cardiac myocytes, with an enhanced basal production of nitric oxide observed in patients with heart failure [16], [17]. Our earlier studies on DCM have shown increased levels of NO in the DCM group compared to the controls [18], which corroborates with an excessive production of nitric oxide by the cardiomyocytes, when exerted over longer periods, leading to reduced myocardial contractility & DCM phenotype [19].

It is hypothesized that eNOS expression and/or activity vary according to the haplotype clusters. However studies could associate a haplotype of eNOS polymorphisms (−786C/4b/Asp298) with hypertension [20], hence haplotype analysis was carried out to identify specific haplotype associated with DCM. Linkage disequilibrium analysis also revealed the loci combinations T-786C:27 bp_VNTR and 27 bp_VNTR:G894T to be in perfect LD (D′ = 0.99, 0.96). Hence it can be stated that the 27 bp VNTR polymorphism is in perfect association with either of the polymorphisms studied, and exhibits its effect synergistically in conjunction with the other polymorphisms, but as a single biomarker its effect on DCM phenotype is negligible. Haplotype analysis revealed, C4bT haplotype combination to be significantly associated with the disease group with 7 folds increased risk to DCM (p<0.0001), further strengthening that these three *NOS3* polymorphisms may be synergistically involved in the etiopathogenesis of DCM. Wang et al 2002 [21], have demonstrated that 27 bp VNTR polymorphism coordinates with –786C variant and regulates transcription efficiency. The homozygotes of G894T in association with at least one C allele of T-786C polymorphism were proposed to be at higher risk for CAD [22]. These findings highlight the synergistic effect of these polymorphisms in haplotype specific fashion.

It is also reported that the genetic heterogeneity of endogenous NO production through NOS3 genetic variations may affect left ventricular remodeling [13], [23], which is an important pathological feature in dilated cardiomyopathy. The detrimental effect of excess NO is through its action on mitochondria via the increased oxidant production resulting in cell death [24]. Hence C4bT may be one such cluster implicated with LV remodeling in DCM.

## Materials and Methods

Blood samples were collected from 115 DCM patients referred to CARE hospitals, Krishna Institute of Medical Sciences (KIMS) and Niloufer hospital for Children, Hyderabad, India. Clinical evaluation was done based on physical examination, chest radiography, electrocardiography and echocardiography. Patients above 25 years of age were subjected to cardiac catheterization and coronary angiogram to exclude the possibility of significant coronary artery disease or valvular disease. Blood samples from 454 age, sex matched randomly selected healthy subjects without any history of cardiac and systemic disorders, served as controls. Samples were collected from the subjects after obtaining written consent. The study was approved by institutional ethical committee of CARE Hospital and Osmania University, Hyderabad. The samples were procured and the genomic DNA was isolated from the blood samples following standard protocols [25], [26].

PCR amplification was carried out to screen for NOS3 polymorphisms using specific primers. The frequency of the T-786C and G894T polymorphisms were determined by polymerase chain reaction (PCR)-restriction fragment length polymorphism (RFLP) as described previously [9], [10] while the 27 bp_VNTR polymorphism of intron 4 was examined by allelic specific PCR. Each PCR was optimized with respect to the Mg2+ ion concentration. The PCR-mix consisted of 10xPCR buffer, 10 µM dNTP-mix, 1 µM of each primer, 1 U Taq-polymerase and 60 ng template DNA in a reaction volume of 10 µl. Reactions were carried out in an Eppendorf Master Cycler Gradient (Germany).

### T-786C Polymorphism (rs2070744)

The 494 bp PCR product was digested with *Msp*I enzyme (Merck, Germany). Digested samples were separated on 12% non-denaturing polyacrylamide gel and visualized by silver staining. The genotypes were identified as TT (258/240 bp), CT (258/240/212/46 bp), and CC (240/212/46 bp) respectively.

### G894T Polymorphism (rs1799983)

The 246-bp PCR product was digested with a *Dpn*II Restriction enzyme (New England Bio labs, USA) and resolved on 10% polyacrylamide gel with silver staining. The presence of T allele was identified on creation of a restriction site for the enzyme (*Dpn*II).

### 27 bp_VNTR Intron 4 Polymorphism

The repeats are of a 27-bp consensus sequence with two alleles resolved on 3% agarose gels, the larger allele of 420 bp has five tandem repeats and designated as ‘b’- insertion, and the smaller allele of 393 bp ‘a’- deletion has four repeats [27].

### Statistical Analysis

Hardy-Weinberg equilibrium was established by adopting the *X^2^* test (Medcalc software), for all polymorphisms in cases and controls. Odds ratios, with 95% confidence intervals were calculated to compare allele and genotype frequencies. The extent of linkage disequilibrium (LD) was expressed in terms of the maximum likelihood estimate of disequilibrium, *D*′. Statistical analysis was carried out using SNPstats software, available online (www.bioinfo.iconcologia.net/SNPstats) and Haploview software. For all tests, significance level was set as p<0.05.

### Conclusions

Epistatic interaction is an important determinant in the susceptibility of complex human diseases. As DCM is a complex disorder with several pathways implicated in the disease pathogenesis, oxidative stress could be one of the possible mechanisms in DCM manifestation. In the present study, the NOS3 variants (T-7869C, G894T) appear to play a significant role in DCM leading to heart failure, with the implication of C4bT haplotype cluster influencing LV remodeling. Hence our findings further strengthen the observation that the DCM is an end result of oxidative stress [18], culminating into heart failure.
